# Polysaccharides from *Brasenia schreberi* with Great Antioxidant Ability and the Potential Application in Yogurt

**DOI:** 10.3390/molecules29010150

**Published:** 2023-12-26

**Authors:** Yujie Wang, Yue Zou, Qiong Fang, Ruizhang Feng, Jihong Zhang, Wanhai Zhou, Qin Wei

**Affiliations:** 1 Faculty of Agriculture, Forestry and Food Engineering, Yibin University, Yibin 644000, China; wangyjsicau@126.com (Y.W.); zouyue255@163.com (Y.Z.); fangfang_0717@163.com (Q.F.); ruizhangfeng@126.com (R.F.); ysl199805@hotmail.com (J.Z.); 2 Sichuan Oil Cinnamon Engineering Technology Research Center, Yibin 644000, China; 3 School of Food and Bioengineering, Xihua University, Chengdu 610039, China; 4 Beijing Advanced Innovation Center for Food Nutrition and Human Health, Beijing Technology and Business University (BTBU), Beijing 100048, China

**Keywords:** *Brasenia schreberi*, enzymatic hydrolysis, polysaccharides, antioxidant ability, polysaccharide yogurt

## Abstract

*Brasenia schreberi* is a widely consumed aquatic plant, yet the knowledge regarding its bioactive components, particularly polysaccharides, remains limited. Therefore, this study aimed to optimize the extraction process of polysaccharides from *B. schreberi* using the response surface method (RSM). Additionally, we characterized the polysaccharides using various methods and assessed their antioxidant capabilities both in vitro and in vivo, employing cell cultures and *Caenorhabditis elegans*. Furthermore, these polysaccharides were incorporated into a unique yogurt formulation. Our findings demonstrated that hot water extraction was the most suitable method for extracting polysaccharides from *B. schreberi*, yielding samples with high sugar content, significant antioxidant capacity, and a well-defined spatial structure. Moreover, pectinase was employed for polysaccharide digestion, achieving an enzymolysis rate of 10.02% under optimized conditions using RSM. Notably, the results indicated that these polysaccharides could protect cells from oxidative stress by reducing apoptosis. Surprisingly, at a concentration of 250 μg/mL, the polysaccharides significantly increased the survival rate of *C. elegans* from 31.05% to 82.3%. Further qPCR results revealed that the polysaccharides protected *C. elegans* by up-regulating the *daf-16* gene and down-regulating mTOR and insulin pathways, demonstrating remarkable antioxidant abilities. Upon addition to the yogurt, the polysaccharides significantly enhanced the water retention, viscosity, and viability of lactic acid bacteria. These outcomes underscore the potential of polysaccharides from *B. schreberi* as a valuable addition to novel yogurt formulations, thereby providing additional theoretical support for the utilization of *B. schreberi*.

## 1. Introduction

*Brasenia schreberi*, a perennial aquatic herb belonging to the Nymphaeaceae family, is currently under the threat of extinction, with its distribution confined to subtropical regions located south of 30° N, including countries such as China, Japan, South Korea, Australia, North America, Cuba, and Mexico [1]. In traditional folklore, *B. schreberi* was used as a dietary salad ingredient. Recent studies have highlighted its wide-ranging properties, demonstrating not only antioxidant and anti-inflammatory attributes but also revealing an inhibitory effect on HIV [1,2]. These findings have sparked significant scientific interest, particularly in the fields of nutritional and pharmaceutical research.

However, it is essential to note certain limitations in the research focused on polysaccharides derived from *B. schreberi*. While polysaccharides are widely recognized as the most abundant and main bioactive constituents in *B. schreberi* [3,4], current research has primarily highlighted their cholesterol-lowering effects and strong antioxidant abilities in vitro [5,6]. Despite these valuable discoveries, further investigations are required to comprehensively understand and harness the full spectrum of potential therapeutic applications associated with these polysaccharides. Future research efforts should aim to explore additional biological activities, elucidate underlying mechanisms, and investigate potential synergistic effects with other bioactive compounds present in *B. schreberi*. Such endeavors may provide a more comprehensive understanding of the diverse health-promoting properties of this botanical species, further enhancing its significance in both medical and nutritional contexts.

Numerous studies have demonstrated that plant polysaccharides have the potential to stimulate the growth of probiotics and regulate the enhancement of intestinal flora [7,8]. Particularly, polysaccharides derived from *Ganoderma lucidum* have been found to significantly increase the abundance of probiotic strains such as *Bifidobacterium*, *Lactobacillus* Johnson, and *Lactococcus lactis* in mice [9]. Likewise, polysaccharides isolated from *Poria cocos* have been shown to reduce the diversity of operational taxonomic units (OTUs) and profoundly remodel the composition of gut microbiota, favoring bacteria associated with anti-obesity effects, short-chain fatty acid production, and lactic acid production [9]. The recent literature has also indicated that the incorporation of polysaccharides during the fermentation process of yogurt can improve its texture and taste [10]. More specifically, polysaccharides can enhance the water-holding capacity of yogurt, fortify the protein-based network structure, and boost the stability of yogurt. Furthermore, the proliferation of lactic acid bacteria is facilitated [11]. Currently, some functional polysaccharides sourced from *Ganoderma lucidum*, *Tricholoma matsutake*, *Pleurotus eryngii*, and Citrus peel have been utilized as additives in yogurt production, yielding a distinctive and nutritious food product [12,13].

Degradation of polysaccharides into small molecular polysaccharides is an effective way to improve bioactivity. Likewise, polysaccharides from *Polygonatum sibiricum* and *Auricularia auricula* were degraded and then the degradation products surprisingly showed a better antioxidant ability [14,15]. Nowadays, due to the high-water solubility, the polysaccharides can be added to make functional fermented yogurt, which is also one of the development trends of the current dairy market [12,13]. At present, the research about polysaccharides from *B. schreberi* is more focused on the extraction method and structure composition [4,16]. Few researchers reported the degradation process of polysaccharides from *B. schreberi*, and the addition of degraded polysaccharides into food to make functional food, as most of their works are focused on the antioxidant ability in vitro [5].

Therefore, in this study, the aim was to explore the optimization process of enzymolysis of the polysaccharide from *B. schreberi*, evaluate the antioxidant ability of the polysaccharide, and to develop a high-quality yogurt with unique flavor and function using the obtained polysaccharide degradation products as an addition. This provided technical and theoretical support for the development of functional polysaccharide yogurt and enriched the research route of products from *B. schreberi*.

## 2. Results and Discussions

### 2.1. Chemical Properties of Polysaccharides Extracted from Different Conditions

As shown in Table 1, the total sugar content in water extraction polysaccharides (W-BSP) was the highest with 60.86% while alkaline extraction polysaccharides (A-BSP) had the least with 35.06%. The content of crude polysaccharides obtained using enzymatic extraction was in the middle with 54.45%, which was slightly lower than that in W-BSP, indicating that the pure enzymatic extraction of polysaccharides could not obtain higher yield, and the combination with other extraction methods might help to improve the yield. In addition, the reducing sugar content of polysaccharides extracted using the enzymatic method was the highest (enzymatic extraction polysaccharides, E-BSP, 1.44%). The three polysaccharides were all obtained after alcohol precipitation, and most reducing sugars in the extract were removed in 80% ethanol solution. Therefore, the reducing sugar content of polysaccharides obtained using the three extraction methods was low. It can be seen from Table 1, the protein content in A-BSP (9.45%) was much higher than that in E-BSP and W-BSP. The three extraction methods of polysaccharides yielded negative results in the iodine-potassium iodide reaction results, and the results of the ferric chloride reaction were also negative, meaning starch and phenolic hydroxyl did not exist in the three polysaccharides.

The polysaccharides obtained using the three extraction methods were tasteless without phenolic hydroxyl and starch. Among them, the total sugar content of A-BSP (38.06%) was significantly lower than that of W-BSP (60.86%). In addition, the protein content (9.45%) was the highest and the color was the darkest. This may be beecause alkaline solution can dissolve not only a large number of neutral polysaccharides and acidic polysaccharides, but also substances that are insoluble in water. Moreover, the Maillard reaction can be promoted under alkaline conditions, resulting in the deepening of the color of the extract and difficult purification.

### 2.2. Triple Helix Structure of Different Polysaccharides

**Figure 1:**
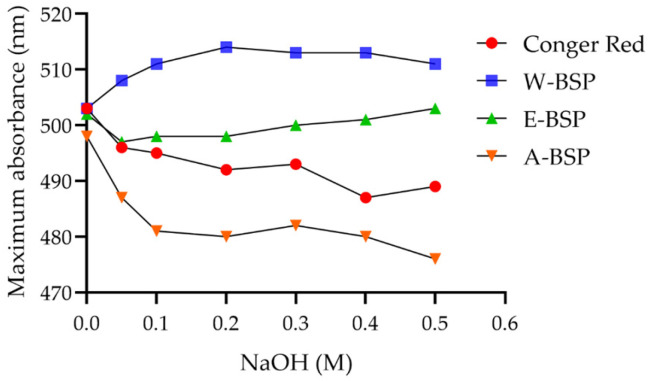
Change in the maximum absorption wavelength of polysaccharides.

Congo red experiment can preliminarily determine whether there is a triple helix structure in polysaccharides [17]. As shown in Figure 1, when the concentration of sodium hydroxide increased gradually from 0 to 0.2 mol/L, the maximum absorption wavelength of W-BSP showed an obvious upward trend and reached the maximum at 0.2 mol/L, showing the red shift. After 0.2 mol/L, the maximum absorption wavelength decreased slowly, indicating that the triple helix structure was destroyed. Obviously, the maximum absorption wavelength of A-BSP was lower than that of the Congo red solution, meaning the triple helix structure did not exist in A-BSP. Although the red shift occurred in E-BSP, there was no downward trend at high concentrations of sodium hydroxide, hence it can be considered that there was no triple helix structure in E-BSP. Hence, the spatial structure of polysaccharides extracted by using hot water extraction was more complete and more suitable as the raw materials for degradation.

### 2.3. The Antioxidant Capacity of Different Polysaccharides

**Figure 2:**
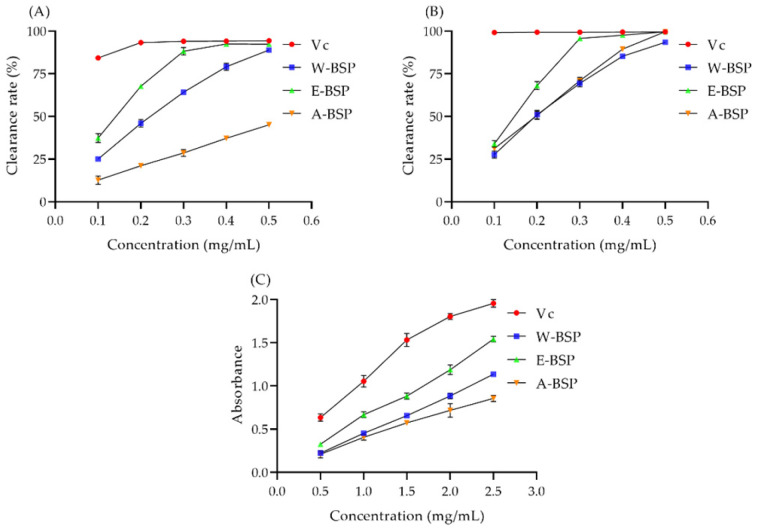
The antioxidant ability of the three polysaccharides. (**A**) The scavenging ability on (**A**) DPPH and (**B**) ABTS free radicals. And (**C**) the total reducing power.

As shown in Figure 2A, the DPPH· and ABTS free radical scavenging capacity was also gradually increased with the increasing concentration of crude polysaccharides. The IC50 values of A-BSP, E-BSP and W-BSP on DPPH were 0.55 mg/mL, 0.16 mg/mL and 0.23 mg/mL, respectively (Figure 2B). Meanwhile, The IC50 values of A-BSP, E-BSP and W-BSP on ABTS were 0.20 mg/mL, 0.15 mg/mL and 0.21 mg/mL, respectively. In addition, the absorbance of the three polysaccharides was increased, indicating an increased reducing power (Figure 2C). These results revealed that W-BSP possesses a strong antioxidant ability. Hence, in the following assays, W-BSP was selected for further enzymatic hydrolysis.

### 2.4. Characteristics of Polysaccharides

**Figure 3:**
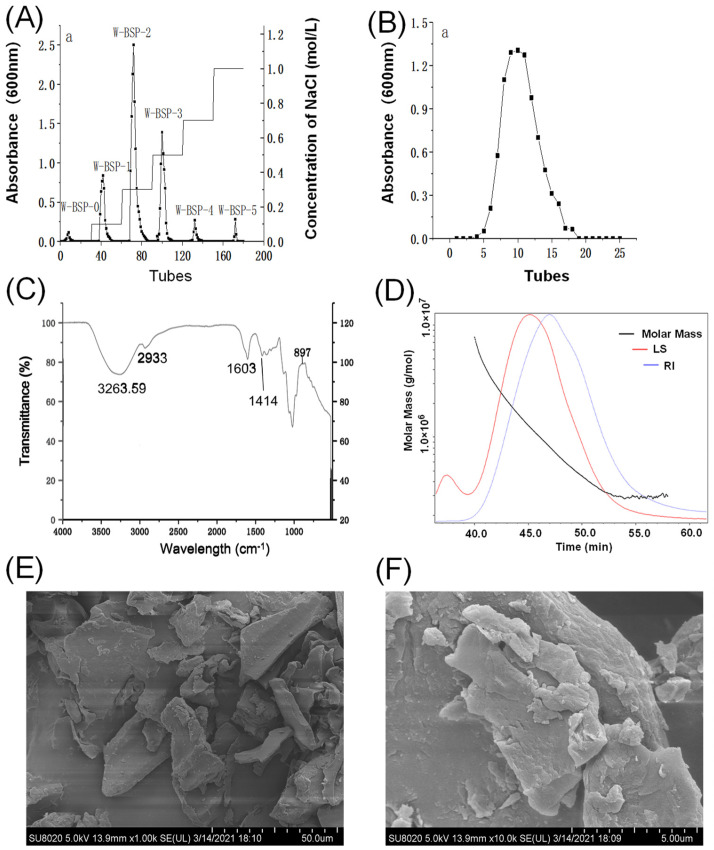
The characteristics of the polysaccharides. (**A**) Separation of the polysaccharides using DEAE Cellulose DE-52 gel; (**B**) separation and purification of Sephadex G-100 gel; (**C**) the Fourier Transform Infrared Spectroscopy spectra of the polysaccharides; (**D**) the GPC-RI-MALLS results of the polysaccharides; (**E**) SEM of the polysaccharides under 1000 magnification; and (**F**) SEM of the polysaccharides under 10,000 magnification.

After DEAE-52 column elution, the W-BSP-2 component accounted for 51.59%, which was the largest component (Figure 3A). After continuing to use the G-100 gel for elution, only one curve can be seen (Figure 3B), indicating that this part of the polysaccharide was a homogeneous polysaccharide. The infrared spectrum showed that the strong absorption peak at 3263.59 cm^−1^ was the result of the O-H stretching vibration of intermolecular hydrogen bonds [18]. The strong absorption peak at 2933 cm^−1^ was the C-H stretching vibration of methyl or methylene [19]. Near 1603 cm^−1^ was the characteristic peak of sugar hydrate [20]. 1414 cm^−1^ was the C-H changing angle vibration absorption peak of sugar (Figure 3C). After GPC-RI-MALLS measurement, the average relative molecular mass of the W-BSP-2 component was 6.19 × 10^5^ (Figure 3D). The weight of the average relative molecular mass was 9.45 × 10^5^. The root mean square radius of the rotation was 42.1 nm, and the weight root mean square (the radius of rotation) was 50 nm, and the polydispersity coefficient of W-BSP-2 was 1.525, indicating that the relative molecular weight distribution of the polysaccharide was narrow, and the molecular structure was relatively uniform. Following HPLC, it was determined that W-BSP-2 contained D-mannose, rhamnose, D-glucuronic acid, D-glucose, D-galactose, L-arabinose, and L-fucose 7 kinds of monosaccharides. The relative molar masses of the seven monosaccharides are as follows: 1; 1.86; 0.90; 0.06; 7.10; 1.39; and 3.82. And the percentages are 6.20%, 11.53%, 5.58%, 0.37%, 44.002%, 8.62%, and 23.68%, respectively. Under a 1000× microscope, the surface of W-BSP-2 was smooth and had a compact structure (Figure 3E). When the scanning electron microscope magnified it to 10,000×, it was observed that the surface of W-BSP-2 had a rough flaky structure with a corrugated structure, and the structure was still compact (Figure 3F).

### 2.5. Optimization of Enzymatic Hydrolysis

#### 2.5.1. The Effect of Single Factors on Enzymatic Hydrolysis

**Figure 4:**
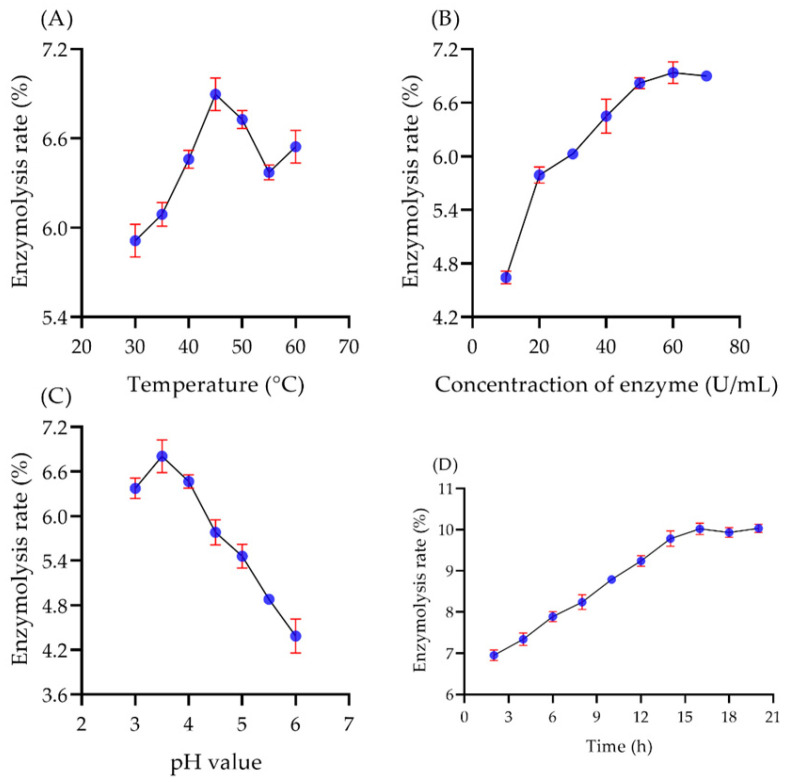
The effect of four single factors on the enzymolysis rate including (**A**) temperature, (**B**) concentration of enzyme, (**C**) pH value, and (**D**) fermentation time.

Five kinds of enzymes were applied to the enzymatic hydrolyze polysaccharide under the optimum conditions, and the results showed that pectinase was the ideal degradation enzyme with the highest enzymatic hydrolysis rate of 4.65% (Supplementary Table S4). As shown in Figure 4A, the degradation rate was increased as the temperature increased, and the highest rate was 6.89% at 45 °C. A high temperature might destroy the structure of the enzymes. Hence 45 °C was selected as the optimum temperature for enzymatic hydrolysis. As the concentration of the enzyme increased to 60 U/mL, the degradation rate was promoted, and the maximum rate was 6.93% (Figure 4B). Continued increase in enzyme concentration did not increase the degradation rate of polysaccharides but lead to more enzyme residues. Therefore, 60 U/mL enzyme concentration was selected as the optimal enzyme concentration for enzymatic hydrolysis. Meanwhile, the optimal pH condition was easily found at 3.5 (Figure 4C). When the reaction time was 16 h, the enzymatic hydrolysis rate fluctuated around 10.02% (Figure 4D). Due to the use of the biological enzyme method for polysaccharide degradation, the conditions were very mild and the reaction speed was slow. Therefore, in the following RSM assay, the time was set at 2 h, and the time was set back at 16 h when the other conditions were optimized.

#### 2.5.2. RSM Analysis

The equation for the enzymatic hydrolysis of polysaccharides was obtained through multivariate linear regression binomial fitting as follows:

Y = 6.799 + 0.519A + 0.004B + 0.055C + 0.034AB + 0.007AC − 0.001BC − 0.196A^2^ − 0.354B^2^ − 0.386C^2^.

The R^2^ was 0.9758, close to 1, meaning the model was significant. Variance results showed that the equation *p* value of the model was lower than 0.05. And the lack of fit was 0.2779 higher than 0.05 which was not significant (Table 2). The *p* value of A, A^2^, B^2^, and C^2^ were lower than 0.05 which was extremely significant. As shown in Supplementary Figure S1, the interaction between enzyme concentration and temperature and pH value had great influence on the degradation rate of the polysaccharide, while the interaction between temperature and pH value had little influence on the degradation rate. According to the F value, the effect of various factors on the degradation of polysaccharides was enzyme concentration > temperature > pH value.

Therefore, the model can be used to predict and analyze enzymatic hydrolysis. The optimal extraction conditions predicted were as follows: temperature was 45.80 °C, pH was 3.53, enzyme concentration was 60 U/mL, and the predicted degradation rate was 7.09%. In order to facilitate the experiment, the conditions were optimized as follows: temperature 45 °C, pH 3.5, and enzyme concentration 60 U/mL. Under these conditions, the average degradation rate of polysaccharides was 6.96%, which was not significantly different from the predicted value. Under the optimal conditions, the hydrolysis time was set back 16 h, and the average hydrolysis rate was 10.02%.

### 2.6. The Antioxidant Ability of Polysaccharides In Vitro and In Vivo

#### 2.6.1. Polysaccharides Increased the Cell Viability under H_2_O_2_

**Figure 5:**
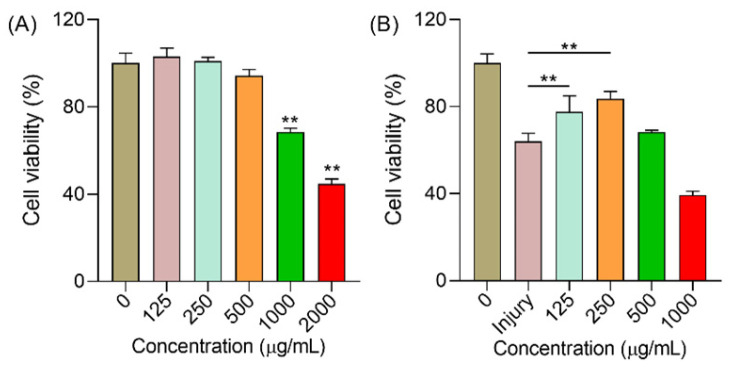
The effect of polysaccharides on HUVEC cells. (**A**) The cell viability of cells after treatment with polysaccharides for 24 h; and (**B**) the cell viability of cells that were exposed to H_2_O_2_. Note: ** means *p* < 0.01.

As shown in Figure 5A, polysaccharides in a high concentration actually had a negative effect on the growth of HUVEC cells. Under 2000 μg/mL conditions, the cell viability was only 44.75% and the viability was 68.45% when the concentration was 1000 μg/mL. Therefore, for the following assays, we chose the lower toxic effect concentration to evaluate the protection of polysaccharides. H_2_O_2_ is an acknowledged trigger for oxidative stress at the cellular level [21,22]. More reactive oxygen species (ROS) will then be accumulated in the body, thus leading to side effects such as ageing, cancer, inflammatory, and so on [23,24,25]. In Figure 5B, the cell viability was only 64.06% when the cells were exposed to H_2_O_2_. When the cells were pre-treated with polysaccharides (125 and 250 μg/mL), the cell viability was significantly increased to 77.60% and 83.66%, respectively, while 500 μg/mL failed to enhance the cell viability (*p* > 0.05). Hence, these results demonstrated that polysaccharides in a low concentration showed low cytotoxicity and can protect cells against oxidative stress.

#### 2.6.2. Polysaccharides Reduced Cell Apoptosis under H_2_O_2_

**Figure 6:**
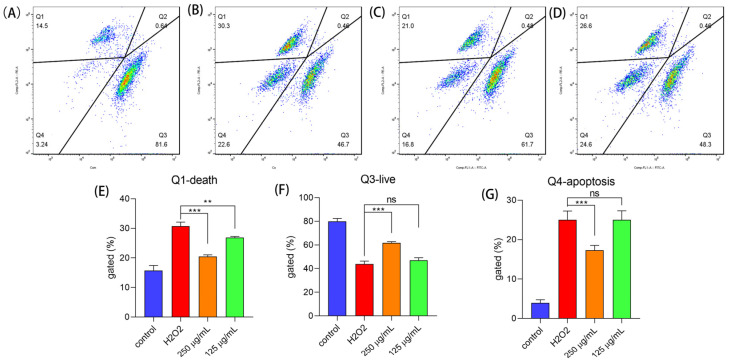
Cell apoptosis determined by AOEB. (**A**) The control group; (**B**) the group that was exposed to H_2_O_2_; (**C**) the group that was treated with 250 μg/mL; (**D**) the group that was treated with 125 μg/mL; and (**E**–**G**) the cell numbers in the Q1, Q3 and Q4 areas, respectively. Notes: *** means *p* < 0.001, ** means *p* < 0.01, and ns means not significant.

Under oxidative stress, cells will suffer cell apoptosis even death [26,27]. In this work, we used AO/EB and AO/PI, two staining methods to assess cell apoptosis. When the cells are alive, EB and PI do not go into the cells and so the PE signal channel is very low, while AO can stain alive cells making the FITC signal channel high. Therefore, the alive cells will be in the Q3 area (Figure 6A), the apoptosis cells will be in the Q4 area, and the dead cells will be in the Q1 area. In Figure 6B, treated with H_2_O_2_, the numbers of cells in Q3 and Q4 were increased. After treatment with polysaccharides, the apoptosis cells were reduced (Figure 6C,D). In the Q1 area, the number of cell deaths was reduced (Figure 6E) and the number of live cells increased after the cells were treated with polysaccharides (Figure 6F); while only 250 μg/mL concentration of polysaccharides could lower cell apoptosis (Figure 6G). Like other natural plants, the bioactive components can reduce cell apoptosis when the cells are exposed to oxidative stress [28,29].

#### 2.6.3. Polysaccharides Reduced ROS under H_2_O_2_

**Figure 7:**
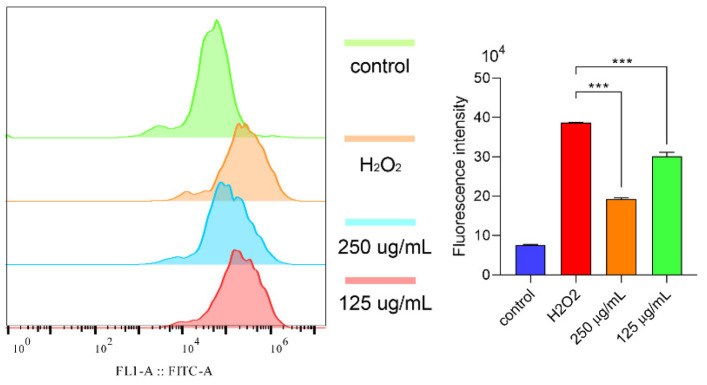
The ROS level in cells detected using flow cytometry. Note: *** means *p* < 0.001.

Then, we also detected the ROS level in the cells. Obviously, H_2_O_2_ could induce a high ROS level (Figure 7). Surprisingly, polysaccharides can lower the ROS level (Figure 6). Other previous works had already proved that H_2_O_2_ caused a high ROS level in cells which then induced a lower cell viability and cell apoptosis resulting in cell death [30]. In this work, we found that polysaccharide treatment can reduce the high ROS level and cell apoptosis. Hence, we drew one conclusion that polysaccharides increased the cell viability against oxidative stress through reducing the ROS level and cell apoptosis, showing a good antioxidant ability in vitro.

#### 2.6.4. Polysaccharides Increased *C. elegans*’ Survival under Thermal Stress

**Figure 8:**
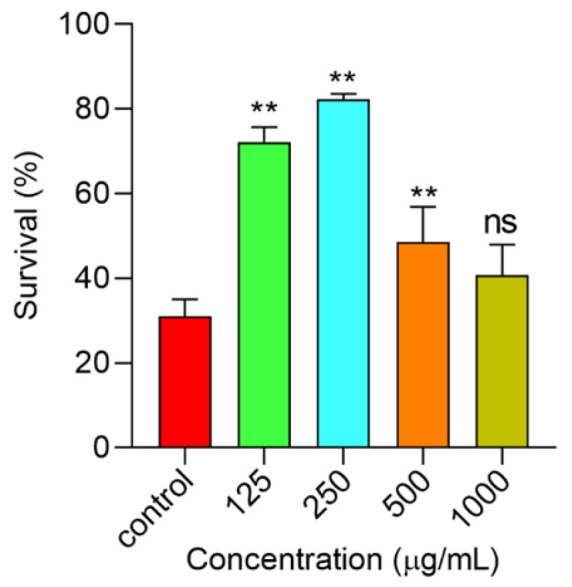
The worm survival under thermal stress. Notes: ** means *p* < 0.01, and ns means not significant.

The *C. elegans* is a good research model to evaluate the pharmacological activity of natural products like polysaccharides, polyphenols, flavone, and so on [30,31,32]. We found polysaccharides showed excellent protection of cells against H_2_O_2_-induced oxidative stress. Hence, the *C. elegans* was applied to further assess the antioxidant ability of polysaccharides in vivo. As shown in Figure 8, the survival rate of the control group was 31.05%. 1000 μg/mL of polysaccharide treatment failed to extend the survival rate (*p* > 0.05), while the other three concentrations could all prolong the survival time of *C. elegans* under thermal stress. Among these, the effect of 250 μg/mL was the best, and the survival rate was increased to 82.3%, which was almost double than that in the control group, while when the concentration of polysaccharides was over 500 μg/mL, especially 1000 μg/mL, the survival of worms was not significantly changed. A high concentration of polysaccharides was also shown to have some toxic effects on HUVEC cells, hence a higher concentration may not be able to increase the survival of *C. elegans* due to the toxicity effect. Others natural polysaccharides also can protect *C. elegans* under thermal stress. Polysaccharides from the shells of *Camellia oleifera* increased the survival rate by 22.69% [30], and Fermented Coix Seed polysaccharides also enhanced the survival by 38.93% [33]. But, compared with the results in this work, polysaccharides exhibited an excellent protection of *C. elegans* under hot conditions.

#### 2.6.5. Polysaccharides Reduced the ROS Level under Thermal Stress

**Figure 9:**
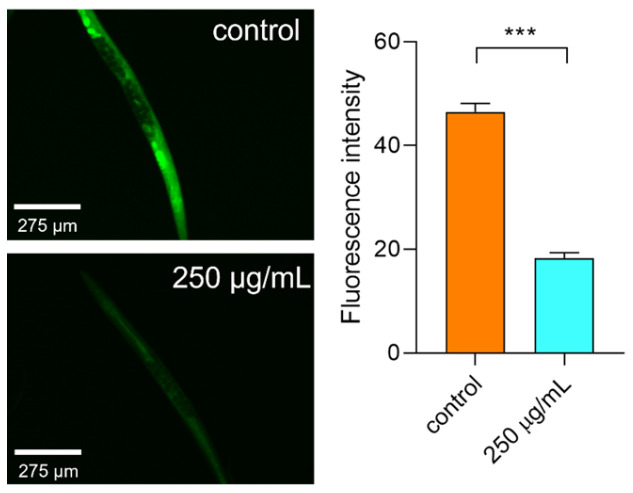
The ROS level in worms under thermal stress. Note: *** means *p* < 0.001.

Thermal stress can cause severe oxidative damage to *C. elegans* even leading to death [32]. Under hot conditions, the ROS level in *C. elegans* was significantly increased. After being treated with polysaccharides, the ROS level was then reduced (Figure 9). Hence, we can say that the polysaccharides can lower the ROS level to relieve the oxidative damage induced by thermal stress.

#### 2.6.6. Polysaccharides Regulated Gene Expression in the IIS/MAPK/mTOR Signal Pathway

**Figure 10:**
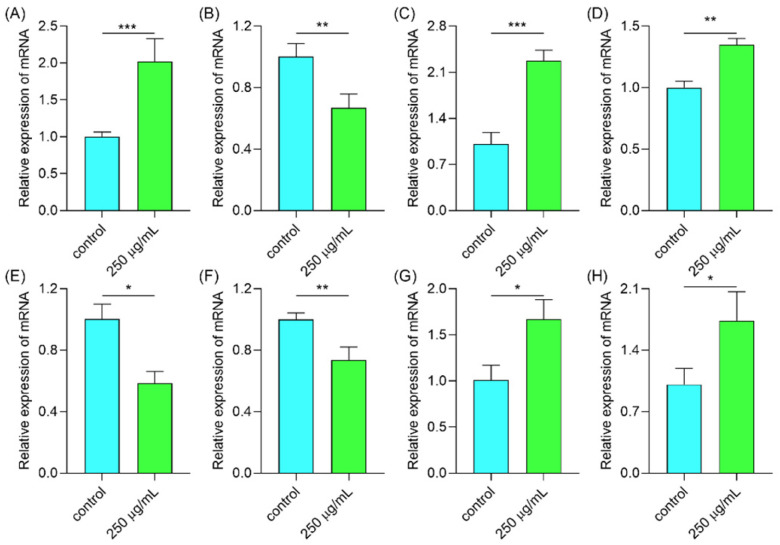
The expressions of genes in worms under thermal stress including (**A**) DAF-16, (**B**) DAF-2, (**C**) HSF-1, (**D**) SKN-1, (**E**) let-363, (**F**) clk-1, (**G**) aak-2, and (**H**) PMK-1. Notes: * means *p* < 0.05, ** means *p* < 0.01, and *** means *p* < 0.001.

Subsequently, we further assessed the expression of some genes that are involved in the defense to stress. For instance, the expression of DAF-16 was significantly increased (Figure 10A). DAF-16 is a key gene that regulates worms to defend stress [34]. While the expression of DAF-16 is regulated by other pathways like the insulin pathway (IIS) [35], surprisingly, the expression of DAF-2 was reduced (Figure 10B), meaning the polysaccharides can down-regulate the IIS pathway. Heat shock factor 1 (HSF-1) is a key transcription factor regulating heat stress and protein folding homeostasis. Therefore, we examined the expression of HSF-1. As shown in Figure 10C, the expression of HSF-1 was obviously increased. Hence the polysaccharides may activate HSF-1 to help worms against stress conditions. SKN-1 is an essential regulator of antioxidant activity and xenobiotic defense [36]. *Glochidion zeylanicum* leaf extracts and *Laminaria japonica* polysaccharide can activate SKN-1 in *C. elegans* under thermal stress to protect the worms [37,38]. In this work, we also found that the expression of SKN-1 was increased (Figure 10D). Moreover, the expressions of let-363 and clk-1 were suppressed (Figure 10E,F), demonstrating that the mTOR signal pathway was involved. The expression of aak-2 was also increased (Figure 10G), meaning the polysaccharides may up-regulate the AMPK signal pathway. PMK-1 is another important gene involved in the core p38 MAPK signaling pathway, and SKN-1 is the downstream target for PMK-1 when the worms respond to stress [39]. As shown in Figure 10H, the expression of pmk-1 was obviously increased. In all, polysaccharides can up-regulate the AMPK and MAPK pathway and down-regulate the IIS/mTOR pathways, and then finally activate DAF-16 to enhance the tolerance ability of *C. elegans*.

#### 2.6.7. Polysaccharides Up-Regulated Gene Expression Involved in the Antioxidant System

**Figure 11:**
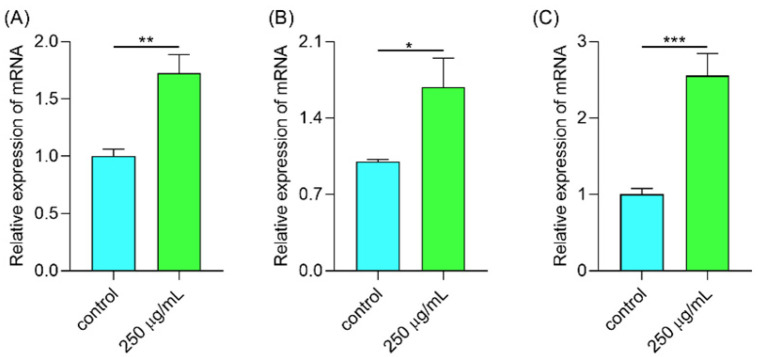
The expressions of antioxidant genes including (**A**) sod-3, (**B**) gst-4, and (**C**) ctl-1. Notes: * means *p* < 0.05, ** means *p* < 0.01, and *** means *p* < 0.001.

Under thermal stress, the ROS level in *C. elegans* was increased. In the organism, the antioxidant system is the main defense system to eliminate the high level of ROS. The sod-3 gene codes for the antioxidant enzyme, sod, in *C. elegans* [40]. Gst-4 and ctl-1 regulate the expression of GSH and CAT enzymes in *C. elegans*, respectively [41]. The above three enzymes are well-known as vital tools to eliminate the exceeding -level of ROS in the organism [42]. Therefore, we further determined the gene expression in the antioxidant system. As shown in Figure 11, the expressions of sod-3, gst-4, and ctl-1 were significantly increased. The three genes were downstream of DAF-16. As the expression of DAF-16 was promoted, the genes downstream of DAF-16 were then activated such as sod-3, gst-4, and ctl1. Hence, we can assume that the polysaccharides activate DAF-16, resulting in a high level of antioxidant enzymes which finally reduces the high ROS.

### 2.7. The Effect of Polysaccharides on Yogurt

#### 2.7.1. Acidity and pH Value of Yogurt

**Figure 12:**
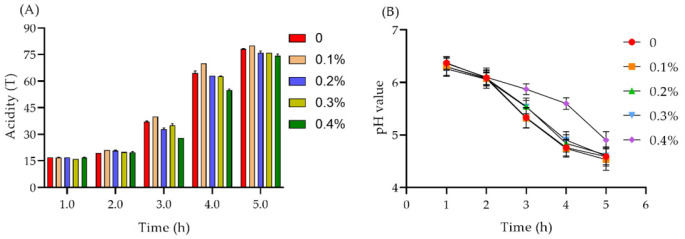
The effect of the addition of polysaccharides on the acidity and pH of yogurt during the process of fermentation. (**A**) the acidity of yogurt after adding polysaccharides for different time. (**B**) the pH changes of yogurt after adding polysaccharides for different time.

The polysaccharide possesses a high antioxidant ability. Hence, we want to add this polysaccharide to make a unique yogurt. Acidity is one of the important physical and chemical indicators that need to be monitored in the production and storage of yogurt. pH value can reflect the acidity of yogurt under certain conditions [43]. As shown in Figure 12A, with the prolonged fermentation time, the titratable acid of fermented milk gradually increased, and it exceeded 70° T after 5 h. When the polysaccharide addition was 0.1%, the titratable acid was boosted, and the pH value decreased (Figure 12B). Therefore, fermentation and acid production was promoted when the polysaccharide concentration was low, while the fermentation of yogurt was inhibited when the added concentration was high.

#### 2.7.2. Water Holding Capacity of Yogurt

**Figure 13:**
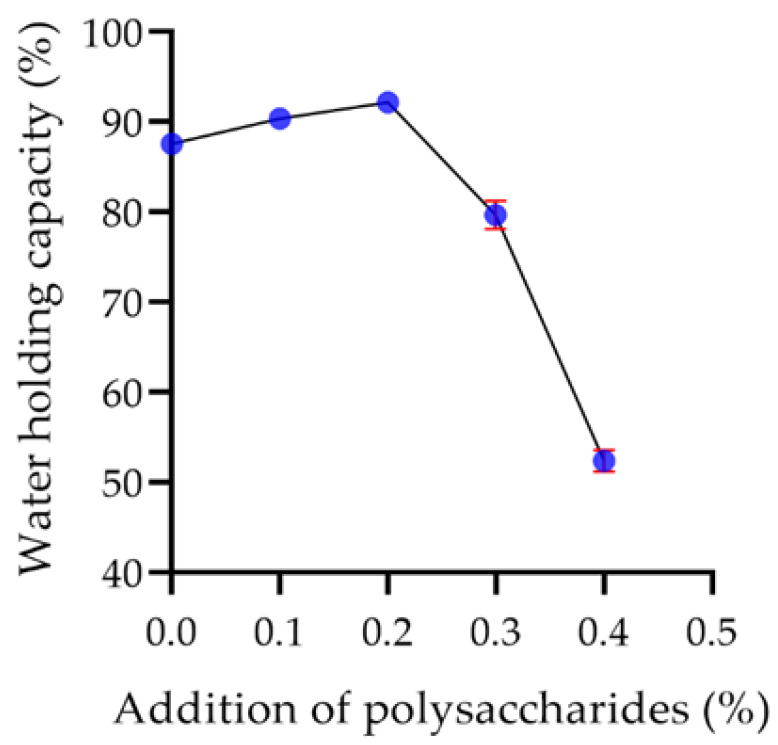
The effect of the addition of polysaccharides on the water holding capacity of yogurt.

The water holding capacity of yogurt is mainly related to its gel structure. The stronger the gel structure is, the higher the water holding capacity is [44]. As shown in Figure 13, the water holding capacity of yogurt enhanced with the increase of polysaccharide concentration. The maximum capacity reached 92.19% when the polysaccharide addition was 0.2%, indicating that a low concentration of polysaccharide can promote the gel structure of yogurt and improve the water holding capacity of yogurt. When the addition of polysaccharides exceeded 0.2%, it was observed that the gel structure of yogurt was inhibited, resulting in a decrease in the water holding capacity of the yogurt.

#### 2.7.3. Viscosity of Yogurt

**Figure 14:**
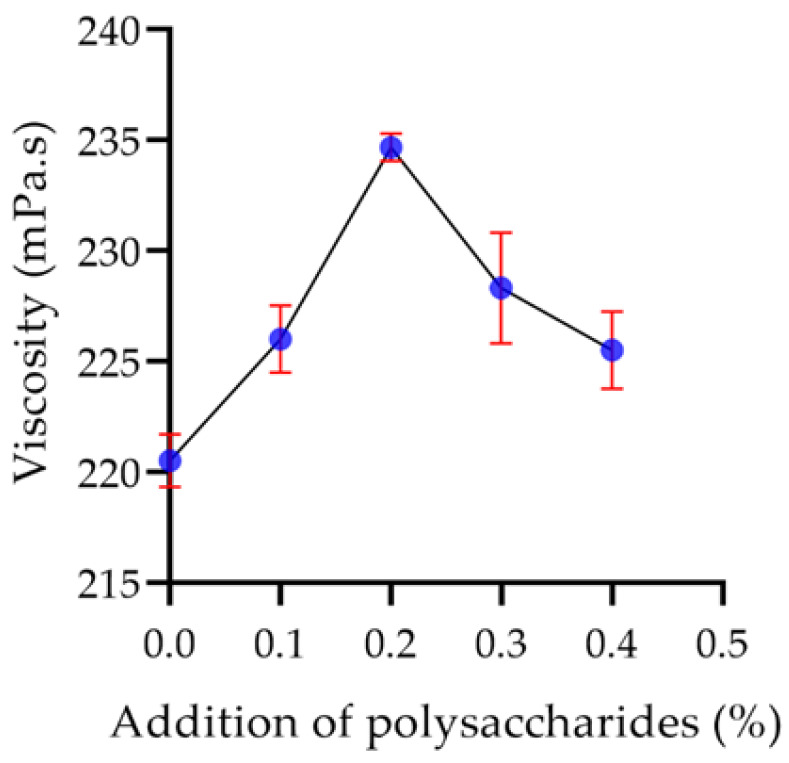
The effect of the addition of polysaccharides on viscosity.

As the polysaccharide concentration increased, the viscosity of the yogurt was promoted. When the polysaccharide concentration was 0.2%, the viscosity reached the maximum value of 234.67 mPa·s (Figure 14). However, when the addition of polysaccharides was more than 0.2%, the viscosity of yogurt decreased. When the addition of polysaccharides exceeds 0.2%, it may disrupt the gel structure of the yogurt, consequently reducing its viscosity.

#### 2.7.4. Texture Property of Yogurt

With the increase of polysaccharide content, the elasticity and adhesion of yogurt increased at first but then decreased (Table 3). Elasticity makes the yogurt more resistant to chewing, and the adhesion makes the yogurt thick and rich in taste [44]. When the polysaccharide content was 0.2%, the elastic measurement value of the yogurt reached the maximum value of 8.94 mm, and when the polysaccharide content was 0.1%, the adhesion measurement value of the yogurt reached the maximum value of 3.02 mj, indicating that the appropriate amount of polysaccharide will increase the elasticity and adhesion of the yogurt. The hardness, adhesiveness and adhesion elongation of the yogurt decreased as more polysaccharide content was added, suggesting that over addition of polysaccharides could weaken the gel structure strength of the yogurt composed of casein.

#### 2.7.5. Viable Lactic Acid Bacteria of Yogurt

*Lactobacillus bulgaricus* and *Streptococcus thermophilus* are the two most commonly used probiotics in yogurt production [45,46]. Probiotics, as a kind of living microorganisms that can give the host health benefits when a sufficient amount is applied, can improve human health by regulating disordered intestinal flora [47]. As shown in Table 4, the number of viable lactic acid bacteria in the polysaccharide yogurt increased significantly compared with the control group. When the polysaccharide content was 0.2%, the number of viable lactic acid bacteria reached the maximum of 7.50 × 10^8^ CFU/mL. And in the above results, we found a higher concentration of polysaccharides showed some toxicity to cells and *C. elegans*, hence, if more polysaccharides were added in, the viability of bacteria may have been affected. Therefore, 0.2% addition is the ideal concentration.

#### 2.7.6. Sensory Evaluation of Yogurt

As shown in Table 5, the sensory score increased as the polysaccharides were added, and the sensory score reached the highest when the added amount was 0.2%, indicating that adding a certain amount of polysaccharide can improve the texture and taste of yogurt. When the added amount was more than 0.2%, the sensory score significantly decreased, meaning that excessive polysaccharides can inhibit the fermentation of yogurt, and affect the coagulation, color and taste of the yogurt. Excessive polysaccharides may react with other components in the milk and inhibit the formation of the three-dimensional gel network structure between casein.

### 2.8. The Effect of Single Factors on Yogurt Quality

#### 2.8.1. Fermentation Temperature

**Figure 15:**
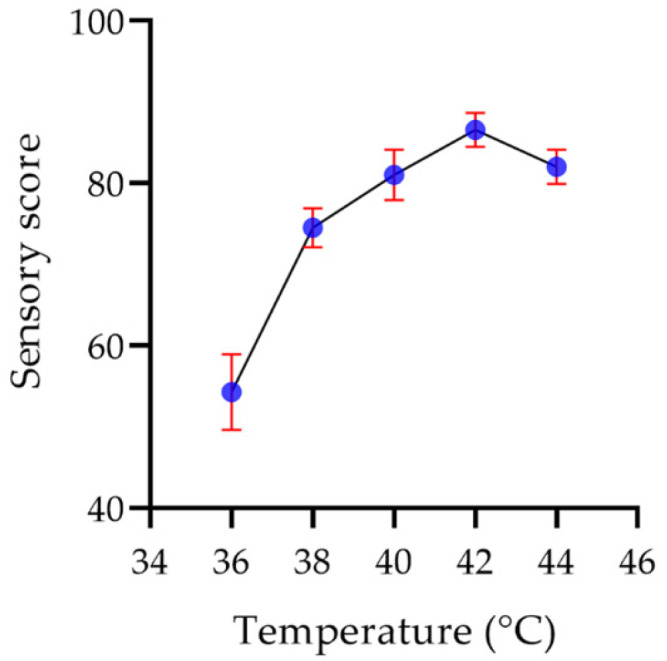
The effect of temperature on the sensory score of the yogurt.

Temperature affects the growth and metabolism of lactic acid bacteria, hence showing a significant impact on the quality of yogurt, mainly on the organizational structure, the flavor and taste of the yogurt, and a little effect on the color [48]. As shown in Figure 15, at a low temperature, the ability of lactic acid bacteria to produce acid decreased, resulting in the failure of coagulation of the yogurt fermented at 36 °C. At 38 °C, the taste of the yogurt was faint, and the flavor of the yogurt was not prominent, resulting in a low score. The yogurt fermented at 40–42 °C coagulated well, the acidity and viscosity of yogurt increased, and the sensory score increased. The sensory score at 42 °C was the highest. However, when the temperature was over 42 °C, the yogurt fermentation was excessive, leading to too much acidity, hence the sensory score was decreased.

#### 2.8.2. Inoculation Amount

**Figure 16:**
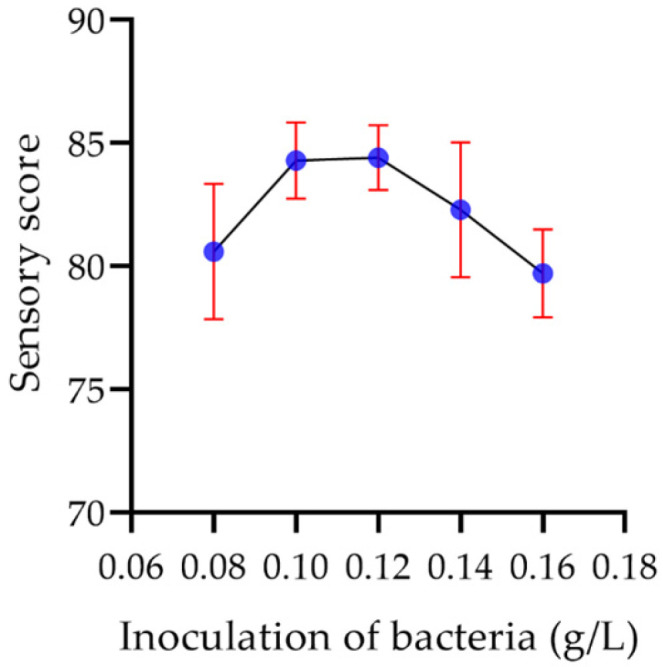
The effect of the inoculation of bacteria on the sensory score of the yogurt.

As shown in Figure 16, compared with other factors, the increase of the inoculation amount of bacterial powder exhibited little effect on the sensory score of the yogurt. When the inoculation amount was 0.10~0.12%, the quality of the yogurt did not change, and the highest sensory score was 84.4 at 0.12%. When the inoculation amount was higher than 0.12%, the sensory score of the yogurt decreased slowly as the inoculation amount increased.

#### 2.8.3. Polysaccharide Concentration

**Figure 17:**
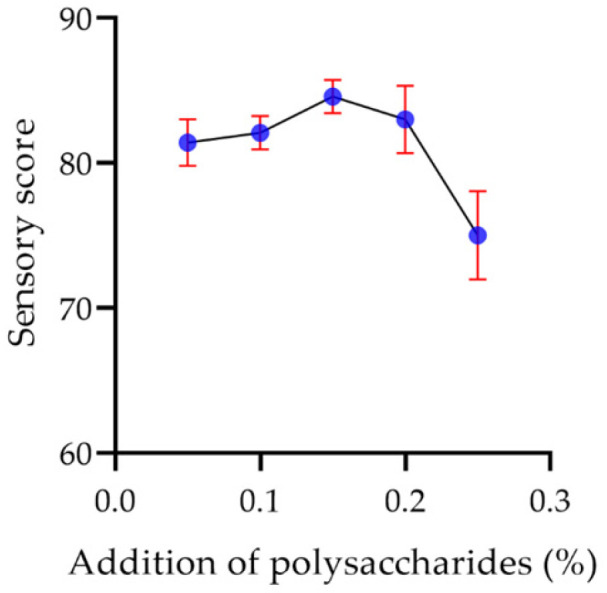
The effect of the addition of polysaccharides on the sensory score of the yogurt.

As the polysaccharide content was increased, the color of the yogurt changed from milk white to slightly yellow, and the taste was gradually viscous. When the polysaccharide concentration was 0.20%, the yogurt began to appear slightly astringent. Figure 17 shows that when the addition of polysaccharide was 0.15%, the color of the yogurt was normal, the taste was delicate, and there was no astringency with a maximum sensory score of 84.6.

#### 2.8.4. Fermentation Time

**Figure 18:**
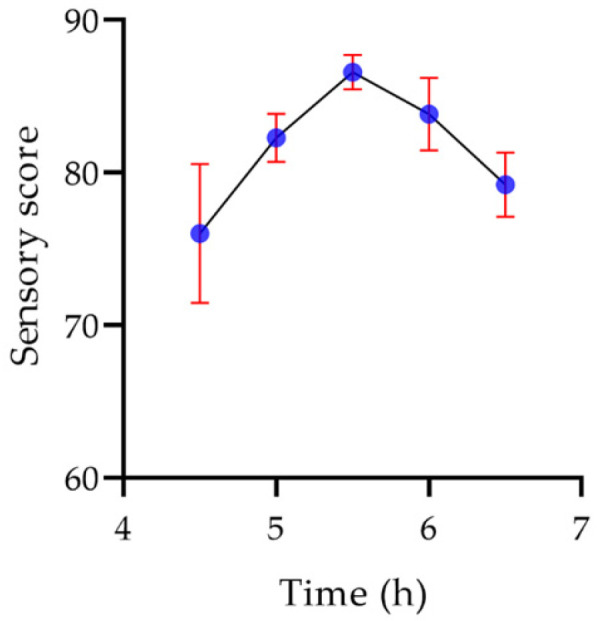
The effect of time on the sensory score of the yogurt.

As shown in Figure 18, the sensory score of the yogurt increased with prolongation of fermentation time. When the fermentation time was short, the yogurt tasted faint and the curd was uneven. However, the yogurt was acidic, and the organization was slightly rough after a longer fermentation time. When the fermentation time was 5.5 h, the yogurt coagulated uniformly and had a delicate taste, and the sensory score of the yogurt was the highest at 86.6.

#### 2.8.5. Sucrose Concentration

**Figure 19:**
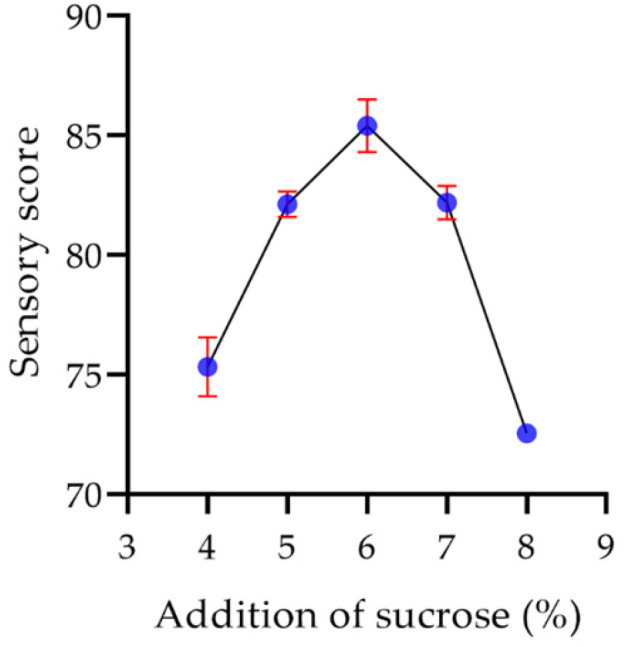
The effect of the addition of sucrose on the sensory score of the yogurt.

In the fermentation of yogurt, the addition of sucrose is mainly to adjust its sweetness, so the addition of sucrose has little effect on the structure, color and flavor of the yogurt [49]. As shown in Figure 19, the sensory score of the yogurt increased as the sucrose content was added. When the sucrose content was low, the yogurt was slightly sour, while the yogurt was too sweet when the sucrose content was high. The sensory score of the yogurt reached the highest value of 85.4 with moderate sweetness when the sucrose content was 6%.

### 2.9. Orthogonal Optimization of Fermentation

Based on the results of single factor assays, three main factors were picked up for the orthogonal test. As shown in Supplementary Table S5, the factors affecting the fermentation of the polysaccharide yogurt were B (sucrose addition) > A (polysaccharide addition) > C (fermentation time). The optimal fermentation combination was selected as A2B2C2: 0.15% polysaccharide, 6% sucrose, and 5.5 h fermentation time.

### 2.10. Quality of Polysaccharide Yogurt after Orthogonal Optimization

Mold and yeast are the two most common types of bacterial contamination found in yogurt. *Staphylococcus aureus* and *Salmonella* are among the most typical food-borne pathogens. These bacteria not only affect the quality of yogurt but can also lead to food poisoning [50]. Therefore, these two pathogens are included as mandatory test items in the national standard for fermented milk. As demonstrated in Table 6, the viable count of lactic acid bacteria in polysaccharide yogurt exceeded 1 × 106 CFU/g, surpassing the threshold outlined in the national standard (China, GB 4789.1-2016) [51]. The counts for molds and yeasts fell within the national standard. Additionally, three other pathogens—*Staphylococcus aureus*, *Salmonella*, and *Escherichia coli*—were not detected in the yogurt samples. Illustrated in Table 7, the water holding capacity, acidity, and protein content of the yogurt were higher than those of regular yogurt, indicating that polysaccharide yogurt has the potential to enhance the nutritional value of yogurt.

### 2.11. Simulated Digestion of Polysaccharide Yogurt In Vitro

The growth of lactic acid bacteria in yogurt in simulated intestinal fluid in vitro is presented in Table 8. The viable count of lactic acid bacteria in both regular yogurt and polysaccharide yogurt exhibited a declining trend at a pH of 1.5, indicating that excessive acidity in artificial gastric juice can impede the growth and reproduction of lactic acid bacteria. At pH levels of 2.5 and 3.5, the viable count of lactic acid bacteria demonstrated a pattern of decrease followed by an increase, suggesting that lactic acid bacteria can gradually adapt to artificial gastric juice at pH 3. Notably, at the same pH level, the number of lactic acid bacteria in polysaccharide yogurt exceeded that in regular yogurt, signifying superior acid resistance of lactic acid bacteria in polysaccharide yogurt compared to regular yogurt. As depicted in Table 9, within artificial simulated intestinal fluid, the viable lactic acid bacteria count in regular yogurt displayed a gradual decline, while the bacterial count in polysaccharide yogurt showed no significant change, indicating a better tolerance of lactic acid bacteria in polysaccharide yogurt to artificially simulated intestinal fluid.

## 3. Materials and Methods

### 3.1. Materials and Reagents

*Brasenia schreberi* JF Gmel. was collected from Leibo Horse Lake, Sichuan Province, China. *B. schreberi* was dried at 40 °C then grounded into fine powder. The fat-soluble substances and pigment were removed using petroleum ether and 95% ethanol. After the powder was decolored and degreased, it was dried at 40 °C again and stored at −80 °C.

Petroleum ether, ascorbic acid (Vc), ethanol, congo red, sulfuric acid, phenol, and other reagents were purchased from Chengdu Kelon Chemical Co., Ltd. (Chengdu, China). Pectinase, glucoamylase, alpha-amylase, cellulase, and papainase were brought from Hefei Bomei Biotechnology Co., Ltd. (Hefei, China). Rose Bengal agar, MC medium, MRS medium, and Baird-Parker agar base were obtained from Guangdong Huankai Microorganism Technology Co., Ltd. (Guangzhou, China).

### 3.2. Polysaccharides from B. schreberi through Different Extraction Methods

Water extraction: Sample powder was mixed with distilled water in the ratio of 1:40 g/mL and set at 80 °C for 2 h with stirring. After repeating the extraction twice, the supernatant was collected.

Alkaline extraction: Sample powder was mixed with sodium hydroxide solution (0.2 M) in the ratio of 1:40 g/mL and set at 80 °C for 2 h with stirring. After repeating the extraction twice, the supernatant was collected.

Enzymatic extraction: Sample powder was mixed with distilled water in the ratio of 1:40 g/mL and cellulase was added into the mixture as 2%. Then, the solution was set at 60 °C for 2 h with stirring. After repeating the extraction twice, the supernatant was collected.

The supernatant was concentrated by a rotary evaporator and 4 volumes of ethanol were added in the concentration at 4 °C for 12 h. After centrifugation, the precipitation was dried at 50 °C and then redissolved in distilled water in the ratio of 4:1000 g/mL at 60 °C with the assistance of the ultrasonic disrupter for 15 min. Subsequently, the solution was centrifugated and the supernatant was concentrated and dialysed for 72 h before lyophilization to obtain 3 different polysaccharides water extractions: polysaccharides (W-BSP), alkaline extraction polysaccharides (A-BSP), and enzymatic extraction polysaccharides (E-BSP).

### 3.3. Assessment of Chemical Property

The total sugar content in the polysaccharides was detected using the phenol–sulfuric acid method [30]. The DNS method was used to detect the content of reducing sugar [52]. The protein content in polysaccharides was detected by the Coomassie brilliant blue method [53]. The iodine–potassium iodide reaction was used to detect whether the polysaccharide contained starch and the presence of phenolic hydroxyl groups in the polysaccharides was detected by the ferric chloride reaction [54,55].

### 3.4. Detection of Triple Helix Structure

The polysaccharides were dissolved in distilled water in the concentration of 1 g/L. 2 mL sample solution was mixed with 2 mL Congo red solution and a different concentration of sodium hydroxide solution. Then, the mixture was placed in the dark for 10 min before the absorbance at 300–600 nm was read.

### 3.5. Determination of the Antioxidant Ability

Three polysaccharides were dissolved in distilled water. Then, the scavenging ability of free radicals was performed according to the previous works [56].

### 3.6. Characterization of the Polysaccharide

Water-extracted polysaccharide (W-BSP) was dissolved in distilled water, then mixed with Sevage solution (chloroform: n-butanol = 4:1, *v*/*v*), and then shaken and centrifuged to remove the denatured proteins. Then, ADS-7 macroporous resin was added for decolorization. The final solution was eluted through the DEAE Cellulose DE-52 ion exchange cellulose column and Sephadex G-100 gel column, and then freeze-dried to obtain water-washed homogeneous polysaccharide.

The polysaccharide sample was mixed with KBr, grinded and pressed into slices, and the functional group was assessed using an infrared spectrum scan in the range of 4000–500 cm^−1^. The monosaccharide composition of the polysaccharide was determined using high-performance liquid chromatography, and a gel chromatography-differential-multi-angle laser light scattering system (GPC-RI-MALLS) was used to determine the molecular weight. SEM electron microscopy was used to scan the surface of the polysaccharide and study its structure.

### 3.7. Preparation of Enzymolysis Polysaccharides

#### 3.7.1. Detection of Enzymatic Hydrolysis Rate

W-BSP was dissolved in distilled water and the total polysaccharides and reducing sugar were determined as A and A0, respectively. The content of reducing sugar was taken as A1 after enzymatic hydrolysis, while the content of reducing sugar in the enzyme was recorded as A2. The enzymatic hydrolysis rate was calculated as the following formula:

Enzymatic hydrolysis rate (%) = (A1 − A0 − A2)/A

#### 3.7.2. Single Factor Experiment on Enzymatic Hydrolysis of Polysaccharides

The polysaccharide sample was dissolved in distilled water in the concentration of 4 mg/mL. Then, five enzymes (pectinase, glucoamylase, α-amylase, cellulase, and papain) were added in at different concentrations ranging from 10 U/mL to 70 U/mL. The pH of the mixture was adjusted by (0.1 M)-citric acid (0.05 M) to 3–6. And the solution was placed at 30–60 °C for 2–20 h. After the water bath, the enzymes were inactivated at 95 °C for 15 min. Then, the reaction mixture was centrifugated at 8000 rpm for 10 min. The content of reducing sugar in the supernatant was assessed and the enzymatic hydrolysis rate was calculated.

#### 3.7.3. Response Surface Methodology for Enzymatic Hydrolysis of Polysaccharides

In order to optimize the enzymolysis process, Box–Behnken design (BBD) in response surface methodology (RSM) was applied to design a three-factor and three-level response surface experiment with the enzymolysis rate as the response value. Temperature, enzyme concentration, and pH were taken as independent variables. The BBD is shown in Supplementary Table S1. Due to the slow reaction velocity of enzymatic hydrolysis, the response surface optimization reaction time was set as 2 h in order to save time and cost. After the optimal conditions were obtained, the experiments were carried out under the optimized conditions of enzymolysis time (16 h) to obtain the maximum enzymolysis rate.

### 3.8. Cellular Assays

#### 3.8.1. Cell Culture

HUVEC cells were obtained from the Institute of Cellular Sciences, Shanghai. Cells were maintained in DMEM medium containing 10% FBS at 37 °C in a 5% CO_2_-incubator. When the cell fusion reached 80%, the cells were digested from the flask for the following assays.

#### 3.8.2. Determination of Cell Toxicity

The HUVEC cells were seeded in a 96-well as the cell density was 8000 cells per well. After 16 h, different concentrations of the polysaccharides (resolved in PBS) were added into each well for a further 24 h incubation. Then, MTT (3-(4,5)-dimethylthiahiazo (-z-y1)-3,5-di- phenytetrazoliumromide) was transferred into the plate for 4 h and the formazan was resolved in DMSO. Finally, the absorbance of the plate was read by a microplate reader at 570 nm [57]. The cell viability was calculated according to the following formula:

Cell viability (%) = 1 − A1/A2(1)

where A1 was the absorbance of the group with polysaccharides; and A2 was the absorbance of the group with PBS instead.

#### 3.8.3. Determination of Cell Protection

The cell suspension was adjusted to 5000 cells per well. After 16 h, polysaccharides were added in the plate for 24 h. Then, the cells were exposed to H_2_O_2_ (100 μM) for 4 h. Finally, the cell viability was detected using the MTT method.

#### 3.8.4. Determination of Cell Apoptosis and ROS Level

The cell density was adjusted to 20,000 cells per well in a 6-well plate. After 16 h incubation, polysaccharides were transferred into the plate for another 24 h. Then, the cells were exposed to H_2_O_2_ for 4 h. Subsequently, the cells were collected in 1.5 mL tubes and washed with cold PBS twice. AO/EB (100 μg/mL) were used to stain the cells for detecting cell apoptosis and DCFH-DA (2’,7’-Dichlorodihydrofluorescein diacetate, 10 μM, from Nanjing Jiancheng Biological Engineering Institute) was used to assess the ROS level through flow cytometry.

### 3.9. In Vivo Assays

#### 3.9.1. *C. elegans* Maintenance

Wide type *C. elegans* was obtained from the *Caenorhabditis* Genetics Center (CGC). The worms were maintained in nematode growth media (NGM) plates with a layer of *Escherichia coli* OP50 as a food source in 20 °C. The synchronized worms were obtained using the bleach method [58]. And the polysaccharides were added into the medium and OP50.

#### 3.9.2. Determination of Survival under Thermal Stress

Synchronized worms were laid down on the NGM plate until they grew to the L4 stage. Then, the worms were transferred to a new plate with or without polysaccharides for 48 h before they were exposed to 35 °C for 7 h. After thermal treatment, the worms were put back in 20 °C for 16 h to count the survival.

#### 3.9.3. Determination of Reactive Oxygen Species (ROS)

After treatment with polysaccharides for 48 h, the worms were put in 35 °C for 4 h. Then, the worms were collected in a 1.5 mL tube and DCFH-DA (100 μM) was added in for 1 h in the dark. Subsequently the worms were washed with K medium 8 times and anesthetized by NaN_3_ (40 mM). Then, the worms were transferred into 96-well plates with a density of 30 worms per well. The fluorescence density was read by a microplate reader and the worms’ ROS level was observed by a fluorescence microscope.

#### 3.9.4. Determination of Gene Expression

After 48 h treatment, the worms were exposed to hot condition for 4 h and then quickly collected and frozen in liquid nitrogen. The worms were then ground with a glass rod. The RNA was extracted according to the RNAeasy™ Animal RNA Extraction Kit (Cat: R0027, Beyotime Biotechnology, Shanghai, China). And the RNA was reversed to cDNA according to the PrimeScript™ RT reagent Kit with gDNA Eraser (Cat: RR047A, Takara Biomedical Technology (Beijing) Co., Ltd., Beijing, China). Then, the qPCR process was performed according to the iTaq™ Universal SYBR^®^ Green Supermix (Cat: 1725124, Bio-Rad Laboratories, Inc., Hercules, CA, USA) in the CFX96 machine (Bio-Rad Laboratories, Inc., Hercules, CA, USA). The data were analyzed by 2^−ΔΔt^ method [59].

### 3.10. Preparation of Polysaccharide Yogurt

The yogurt starter (*Lactobacillus bulgaricus*, *Streptococcus thermophilus*) was purchased from Angel Yeast Co., Ltd. (Hubei, China). The total polysaccharides sample was obtained after enzymatic hydrolysis and dried. The fermentation process was as follows: (A), polysaccharides were dissolved in pure milk at 60 °C; (B), sucrose was then added in for 6% (*w*/*v*); (C), the whole fermentation substrate was sterilized at 90 °C for 15 min; (D), the bacterial powder was inoculated at the concentration of 1.2 g/L after the mixture was cooled to 44 °C; (E), then the mixture was fermented at 42 °C for 5 h; and (F), finally, the yogurt product was obtained after ripening at 4 °C for 24 h.

#### 3.10.1. Determination of Acidity in the Yogurt Fermentation Process

During the fermentation process, the fermented milk was taken out in a conical flask with 20 mL deionized water. Then, 2 mL phenolphthalein indicator was added in. After mixing, the mixture was titrated with sodium hydroxide (0.1 M) until slightly red, without fading, within 5 s. The titration acidity was defined as the volume of sodium hydroxide solution multiplied by 10.

#### 3.10.2. Determination of the pH Value in the Yogurt Fermentation Process

During the fermentation process, the fermented milk was taken out to assess the pH value using a pH meter at 0 h, 1 h, 2 h, 3 h, 4 h, and 5 h.

#### 3.10.3. Determination of the Water Holding Capacity of Yogurt

The yogurt was put in an empty tube (the weight of the tube was recorded as W1). And the total weight of the yogurt with the tube was W2. After centrifugation (3000 r/min, 10 min) and the removal of water, the residual matter was weighed as W3. The water holding capacity was calculated according to the following formula:

Water holding capacity (%) = (W2 − W3)/(W2 − W1)

#### 3.10.4. Measurement of Viscosity

A viscosimeter was applied to assess the viscosity of the yogurt. The post-ripened yogurt (10 g) was put in a tube, then the rotor F1 was stretched with the speed of 60 RPM. The data was recorded at 60 s, 90 s, and 120 s. The average value of the three times was taken as the final viscosity.

#### 3.10.5. Determination of the Yogurt Textural Property

Texture profile analysis of the post-ripened yogurt was carried out using a texture analyzer with a 30 mm probe. The pre-test speed, the test speed, and the post-test speed all were 1 mm/s, and the measurement distance was 25 mm. The textural property was determined including Hardness (N), Elasticity (mm), Glueyness (N), Adhesivity (mj), and Adhesion elongation (mm).

#### 3.10.6. Determination of Viable Count of Lactic Acid Bacteria

The yogurt was resolved in sterilized saline water in a shaken culture box. Then, the solution was diluted to 10^−6^–10^−9^ four gradient dilution. 1 mL dilution was evenly spread on the MRS medium, and incubated at 36 °C in an anaerobic environment for 72 h. Finally, the colonies on the plates were recorded between 30 CFU/mL and 300 CFU/mL.

#### 3.10.7. Sensory Evaluation Score

A total of 20 students and teachers majoring in food scored 100 points according to their taste, and the organizational structure, color, and flavor. The score details are shown in Supplementary Table S2.

### 3.11. Optimization of the Fermentation Conditions

#### 3.11.1. Single Factor Assays

The polysaccharide sample was added into pure milk in the concentration of 0.05–0.25% (*w*/*v*), and sucrose was then added at 5–7% (*w*/*v*). After being fully stirred, different inoculation amounts of bacterial powder were subsequently transferred in (0.8–1.6 g/L). Then, the fermentation matrix was placed at 36–44 °C for 4.5–6.5 h to obtain the polysaccharide yogurt.

#### 3.11.2. Optimization of Fermentation Conditions of Polysaccharide Yogurt by the Orthogonal Experiment

The fermentation temperature was set at 42 °C, the inoculation amount of bacteria was 1 g/L, and the sensory evaluation was directly applied as the evaluation index. An L9 (34) orthogonal experiment design was carried out on the three factors of polysaccharide addition, sucrose addition, and fermentation time. The design is shown in Supplementary Table S3.

#### 3.11.3. Determination of the Indicators of the Polysaccharide Yogurt

Based on the results of orthogonal experiment, the polysaccharide yogurt was prepared using the optimized conditions. Then, the microorganisms were assessed including Lactic acid bacteria, *Escherichia coli*, *Staphylococcus aureus*, *Salmonella*, moulds, and yeasts according to the previous work [60]. In addition, the physical and chemical indexes were detected which involved acidity, protein content, fat, and water holding capacity [10,61].

### 3.12. Simulated Digestion of Polysaccharide Yogurt In Vitro

#### 3.12.1. Production of Polysaccharide Yogurt

The polysaccharide was dissolved in pure milk at a concentration of 0.15% (*w*/*v*) at 60 °C. Subsequently, 6% sucrose was added to the solution, which was then sterilized at 90 °C for 20 min. After cooling to approximately 44 °C, 0.1 g of composite bacterial powder was incorporated, and the entire mixture was incubated at 42 °C for 5.5 h. Following this, the mixture underwent post-ripening at 4 °C, resulting in the production of polysaccharide yogurt.

#### 3.12.2. Simulated Gastric Juice Digestion In Vitro

Pepsin (1%) was completely dissolved in hydrochloric acid solution at varying pH levels of 1.5, 2.5, and 3.5. Subsequently, the solution was filtered to acquire an in vitro simulated gastric juice. A specific quantity of polysaccharide yogurt was introduced into the simulated gastric juice, and the reaction was incubated at 37 °C on a shaking table operating at 100 r/min. The viable count of lactic acid bacteria was enumerated following 0, 1, 2, and 3 h of incubation.

#### 3.12.3. Simulated Intestinal Fluid Digestion In Vitro

Ten grams of Trypsin was dissolved in distilled water to obtain solution A. Subsequently, 6.8 g of potassium dihydrogen phosphate was dissolved in 500 mL of water, and the pH was adjusted to 6.8 using 0.1 mol/L sodium hydroxide solution to obtain solution B. Following this, the two solutions were combined and diluted to a final volume of 1000 mL with distilled water. The resultant solution was then filtered using a 0.22 μm microporous membrane to obtain simulated intestinal fluid for in vitro use. The polysaccharide yogurt was introduced into the simulated intestinal fluid and incubated at 37 °C on a shaking table rotating at 100 r/min. Viable counts of lactic acid bacteria were enumerated in the culture medium at 0, 1, 2, and 3 h.

### 3.13. Data Analysis

The data obtained in this work was analyzed by SPSS 22.0 software and were expressed as mean ± standard deviation. Graphpad Prism 8.0 was applied for drawing.

## 4. Conclusions

The hot water extraction method was found to be particularly suitable for extracting polysaccharides from *B. schreberi* (W-BSP) due to its high sugar content, excellent antioxidant activity, and triple helix structure. Following the enzymatic hydrolysis of W-BSP with pectinase, the resulting polysaccharides exhibited strong antioxidant properties both in vitro and in vivo. Notably, at a concentration of 250 μg/mL, the polysaccharides significantly increased the survival rate of *C. elegans* from 31.05% to 82.3%. Subsequently, these polysaccharides were introduced into yogurt fermentation, revealing that at an additional level of 0.2%, they demonstrated optimal water holding capacity, viscosity, elasticity, and sensory attributes. Furthermore, this addition promoted the proliferation of live lactic acid bacteria. In simulated in vitro gastrointestinal fluid digestion assays, the number of viable lactic acid bacteria in polysaccharide-enriched yogurt was higher compared to normal yogurt. These findings suggest that polysaccharides derived from *B. schreberi* represent a promising addition to yogurt, offering unique food functionality.

## Figures and Tables

**Table 1:** Comparison of polysaccharide yield using different extraction methods.

Chemical Properties	Polysaccharides Extracted Using Different Methods		
	A-BSP	E-BSP	W-BSP
Total sugar content (%)	35.06 ± 0.12	54.45 ± 0.50	60.86 ± 0.41
Reducing sugar content (%)	0.22 ± 0.01	1.44 ± 0.02	0.36 ± 0.01
Protein content (%)	9.45 ± 0.18	1.56 ± 0.01	1.42 ± 0.03
Starch	-	-	-
Phenolic hydroxyl	-	-	-

Note: - means not detected.

**Table 2:** Variance analysis of the regression equation.

Source	Square	Degree of Freedom	F Value	*p* Value
Model	3.65	9	31.39	<0.0001
A	2.16	1	167.12	<0.0001
B	1.21 × 10^−4^	1	9.39 × 10^−3^	0.9255
C	0.025	1	1.9	0.2104
AB	4.63 × 10^−3^	1	0.36	0.5683
AC	1.93 × 10^−4^	1	0.015	0.906
BC	6.90 × 10^−6^	1	5.34 × 10^−4^	0.9822
A^2^	0.16	1	12.6	0.0093
B^2^	0.53	1	40.96	0.0004
C^2^	0.63	1	48.61	0.0002
Lack of fit	0.053	3	1.85	0.2779

**Table 3:** Effect of polysaccharide addition on yogurt texture characteristics.

Addition of Polysaccharides (%)	Hardness (N)	Elasticity (mm)	Glueyness (N)	Adhesivity (mj)	Adhesion Elongation (mm)
0	1.19 ± 0.005 ^a^	6.93 ± 0.38 ^a^	0.51 ± 0.012 ^a^	2.68 ± 0.01 ^a^	10.0 ± 0.089 ^a^
0.1	1.15 ± 0.015 ^b^	8.79 ± 0.08 ^b^	0.50 ± 0.007 ^a^	3.02 ± 0.04 ^b^	5.90 ± 0.030 ^b^
0.2	1.11 ± 0.006 ^c^	8.94 ± 0.07 ^b^	0.49 ± 0.003 ^b^	2.82 ± 0.025 ^c^	4.17 ± 0.032 ^c^
0.3	0.98 ± 0.005 ^d^	7.27 ± 0.16 ^c^	0.44 ± 0.006 ^c^	2.30 ± 0.032 ^d^	3.31 ± 0.036 ^d^
0.4	0.89 ± 0.01 ^e^	5.87 ± 0.10 ^d^	0.41 ± 0.002 ^d^	2.18 ± 0.017 ^e^	2.78 ± 0.043 ^e^

Note: different letters mean different. In the same column, the different letters mean the significance like the value labeled with a is significantly different from that labeled with b.

**Table 4:** Effect of polysaccharide addition on the number of lactic acid bacteria.

Addition of Polysaccharides (%)	Lactic Acid Bacteria (CFU/mL)
0	4.63 × 10^8^ ± 0.35 ^a^
0.1	6.82 × 10^8^ ± 0.21 ^b^
0.2	7.50 × 10^8^ ± 0.10 ^c^
0.3	6.20 × 10^8^ ± 0.17 ^d^
0.4	5.37 × 10^8^ ± 0.36 ^e^

Note: different letters mean significant. In the same column, the different letters mean the significance like the value labeled with a is significantly different from that labeled with b.

**Table 5:** Effect of polysaccharide addition on the sensory quality of yogurt.

Addition of Polysaccharides (%)	Sensory Evaluation	Scores
0	Uniform coagulation, moderate sweet and sour, milky white color, good taste, smell coordination	78.6 ± 1.18 ^a^
0.1	Uniform coagulation, moderate sweet and sour, milky white color, thick taste, smell coordination	80.67 ± 1.64 ^a^
0.2	Uniform coagulation, moderate sweet and sour, structure even, light yellow color, delicate and smooth taste, harmonious odor	81.11 ± 0.90 ^a^
0.3	Coagulation milk, moderate acidity and sweetness, slightly yellow color, good taste, harmonious odor	65.89 ± 1.10 ^b^
0.4	No coagulation, sweet and sour, slightly yellow in color, poor taste	43.89 ± 3.54 ^c^

Note: different letters mean significant. In the same column, the different letters mean the significance like the value labeled with a is significantly different from that labeled with b.

**Table 6:** Bacterium standards of polysaccharide yogurt.

Strains	Standards	Determination
Lactic acid bacteria	≥1 × 10^6^ CFU/g	6.9 × 10^8^
*Escherichia coli*	n = 5, c = 2, m = 1 CFU/g, M = 5 CFU/g	0
*Staphylococcus aureus*	0/25 g	0
*Salmonella*	0/25 g	0
Mold	≤30 CFU/g	8
Yeast	≤100 CFU/g	35

**Table 7:** Physical and chemical standards of polysaccharide yogurt.

Properties	Polysaccharides Yogurt	Ordinary Yogurt
Protein (g/100 g)	2.82 ± 0.04 ^ns^	2.61 ± 0.03
Fat (g/100 g)	3.35 ± 0.02 ^ns^	3.32 ± 0.02
Water holding capacity	91.67 ± 0.24% *	88.90 ± 0.4%
Acidity (°T)	80.15 ± 2.17 *	74.32 ± 1.21
Sensory score	85.23 ± 2.37 *	81.48 ± 1.04

Note: ns means not significant and * means *p* < 0.05.

**Table 8:** Growth of *lactobacillus* in yogurt in vitro gastric digestion.

pH Value	Time (h)	Lactic Acid Bacteria (CFU/mL)	
		Ordinary Yogurt	Polysaccharide Yogurt
1.5	0	3.23 × 10^8^ ± 0.22	3.61 × 10^8^ ± 0.09
	1	3.01 × 10^8^ ± 0.30	3.44 × 10^8^ ± 0.14
	2	1.38 × 10^8^ ± 0.15	2.42 × 10^8^ ± 0.12
	3	1.19 × 10^8^ ± 0.21	2.09 × 10^8^ ± 0.45
2.5	0	4.17 × 10^8^ ± 0.33	5.11 × 10^8^ ± 0.27
	1	3.64 × 10^8^ ± 0.54	3.82 × 10^8^ ± 0.15
	2	4.02 × 10^8^ ± 0.19	4.51 × 10^8^ ± 0.39
	3	5.09 × 10^8^ ± 0.60	5.64 × 10^8^ ± 0.25
3.5	0	4.24 × 10^8^ ± 0.22	5.00 × 10^8^ ± 0.65
	1	3.67 × 10^8^ ± 0.53	4.39 × 10^8^ ± 0.38
	2	4.52 × 10^8^ ± 0.27	4.63 × 10^8^ ± 0.13
	3	4.81 × 10^8^ ± 0.16	5.11 × 10^8^ ± 0.26

**Table 9:** Growth of lactobacillus in yogurt in vitro intestinal digestion.

Time (h)	Lactic Acid Bacteria (CFU/mL)	
	Ordinary Yogurt	Polysaccharide Yogurt
0	6.11 × 10^8^ ± 0.37	6.53 × 10^8^ ± 0.25
1	5.93 × 10^8^ ± 0.54	6.32 × 10^8^ ± 0.18
2	5.62 × 10^8^ ± 0.41	6.38 × 10^8^ ± 0.28
3	5.69 × 10^8^ ± 0.12	6.41 × 10^8^ ± 0.45
4	5.31 × 10^8^ ± 0.14	6.51 × 10^8^ ± 0.30

## Data Availability

Data are contained within the article and supplementary materials.

## Supplementary Materials

The following supporting information can be downloaded at: https://www.mdpi.com/article/10.3390/molecules29010150/s1, Figure S1: The interaction of three factors on the enzymatic hydrolysis rate, including (A) the interaction of temperature and concentration of enzyme, (B) the interaction of pH value and temperature and (C) the interaction of pH value and concentration of enzyme.; Table S1: Box-Behnken design of the response surface method; Table S2: Sensory evaluation standards for polysaccharide yoghurt; Table S3: Factors and levels of orthogonal experiment of polysaccharide yoghurt; Table S4: Screening of degrading enzymes; Table S5: Results of orthogonal experiment.
