# Supplementary material for: Polysaccharides from Brasenia schreberi with Great Antioxidant Ability and the Potential Application in Yogurt
Source: Molecules. 2023 Dec 26;29(1):150. doi: 10.3390/molecules29010150 (PMC10780003; doi:10.3390/molecules29010150)
Supplement: Supplementary file 1 [file molecules-29-00150-s001.zip › molecules-2698267-supplementary.pdf]

Supplementary Table 1 Box-Behnken design of the response surface method

Runs	Concentration of enzyme (A)	pH value (B)	Temperature (C)	Enzymatic hydrolysis rate (%)
1	55	3.5	60	6.69711
2	35	3	50	5.96157
3	45	4	60	6.83077
4	45	3.5	50	6.68243
5	55	3	50	6.04208
6	45	3.5	50	6.80991
7	35	4	50	5.99912
8	45	3.5	50	6.90263
9	55	4	50	6.07437
10	45	3.5	50	6.66852
11	45	3	40	5.65492
12	45	4	40	5.56753
13	45	3	60	6.7821
14	35	3.5	60	6.53949
15	45	3.5	50	6.73574
16	55	3.5	40	5.80086
17	35	3.5	40	5.67106

Supplementary Table 2 Sensory evaluation standards for polysaccharide yoghurt

item	marking criterion	score
taste	soft taste, no graininess, moderate sweet and sour	20
organizational structure	uniform curd, fine texture, no cracks, no whey precipitation	20
color	uniform color, milky white and yellowish	20
flavor	a strong aroma, a harmonious smell, and no peculiar smell	20
favorite	like it very much	20

Supplementary Table 3 Factors and levels of orthogonal experiment of polysaccharide yoghurt

level	Addition of polysaccharides %(A)	Addition of sucrose %(B)	Fermentation time h(C)
1	0.1	5	5
2	0.15	6	5.5
3	0.2	7	6

Supplementary Table 4 Screening of degrading enzymes

Kinds of enzymes	pH	Temperature (℃)	enzymolysis rate （%）
pectinase	4	50	4.65±0.15
diastatic enzyme	4.5	60	4.1±0.06
a-amylase	6.4	60	2.32±0.11
cellulase	5	60	1.29±0.04
Papain protein enzyme	6	55	0.46±0.01

Supplementary Table 5　Results of orthogonal experiment

Runs	Addition of polysaccharides (A)	Addition of sucrose (B)	Fermentation time (C)	Error list	Sensory score
1	1	1	1	1	79
2	1	2	2	2	84
3	1	3	3	3	81
4	2	1	2	3	82
5	2	2	3	1	86
6	2	3	1	2	81
7	3	1	3	2	80
8	3	2	1	3	83
9	3	3	2	1	81
K1	81.33	80.33	81.00	82	
K2	83.00	84.67	82.33	81.67	
K3	81.33	81.00	82.33	82	
k1	27.11	26.78	27	27.33	
k2	27.67	28.22	27.44	27.22	
k3	27.11	27	27.44	27.33	
R	1.67	4	1.33	0.33	

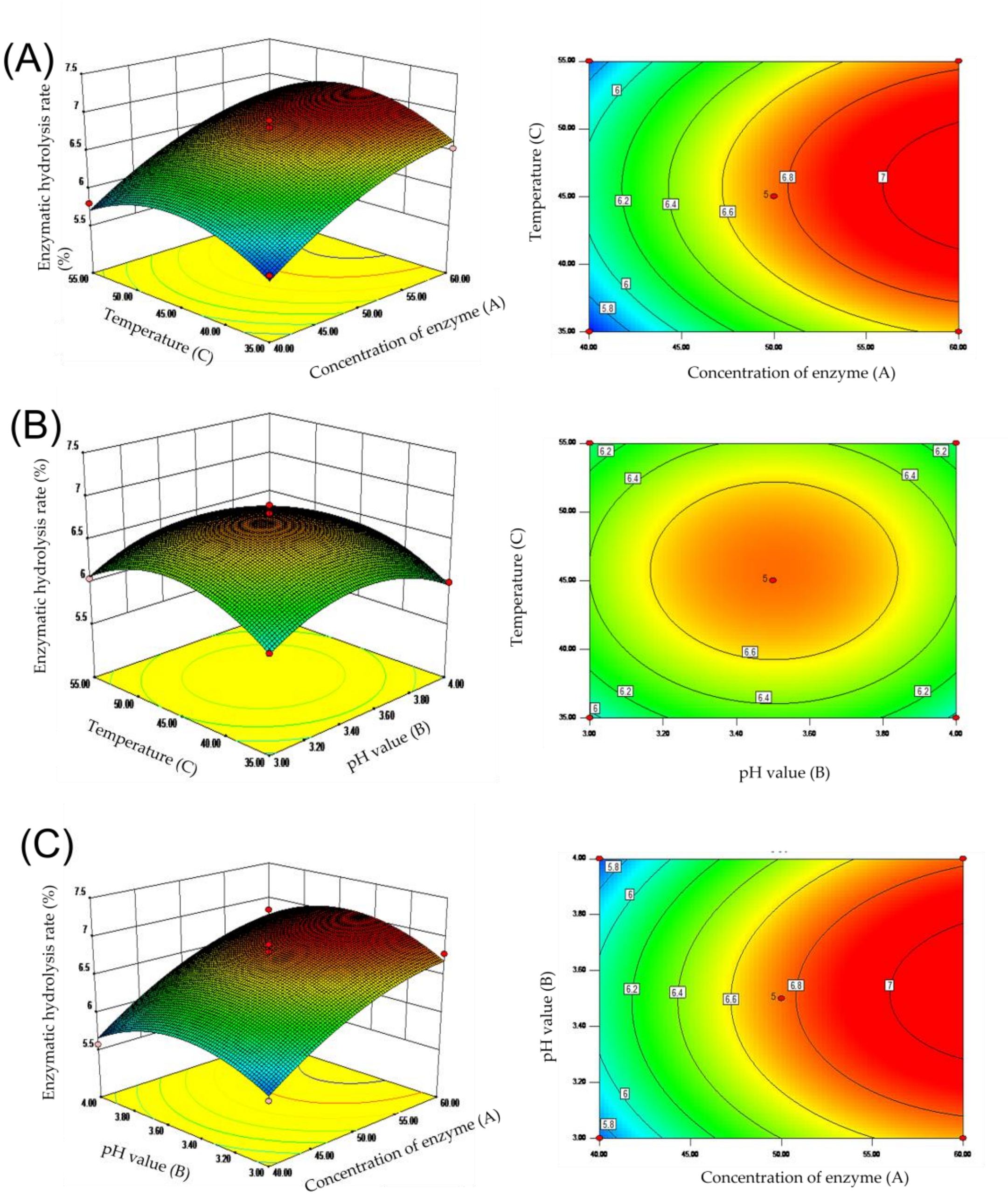

Supplementary Figure 1. The interaction of three factors on the enzymatic hydrolysis rate, including (A) the interaction of temperature and concentration of enzyme, (B) the interaction of pH value and temperature and (C) the interaction of pH value and concentration of enzyme.
